# A Graduated Responsibility Supervising Resident Experience Using Mastery Learning Principles

**DOI:** 10.15694/mep.2019.000203.2

**Published:** 2020-06-30

**Authors:** Benjamin Schnapp, Aaron Kraut, Ciara Barclay-Buchannan, Mary Westergaard, Ciara Barclay-Buchanan

**Affiliations:** 1 University of Wisconsin

**Keywords:** graded, graduated, progressive, supervising, attending, responsibility, roles, residency, GME, emergency

## Abstract

This article was migrated. The article was marked as recommended.

**Introduction:** Supervising other residents and independently running an emergency department (ED) is different from anything residents are asked to do in their early years of residency. There is often little training for this new responsibility. We created a new supervising experience that allows residents to progressively master skills associated with independently running an ED.

**Methods:** We created an experience where PGY-3 residents supervised at our community site, removing the difficulties associated with an academic site such as junior residents and multiple consultants but requiring them to perform all other duties expected of the attending. Residents were scheduled in blocks of 4 shifts to allow them to iteratively improve their performance. Mastery was defined as 23 of 24 points across two shift evaluations, with 3 points available across 4 domains of Organization, Leadership/Communication, Flow and Patient Care. Residents who achieved this standard were allowed to progress to a more advanced supervising experience.

**Results:** Eighty three percent of PGY-3 residents (10/12) achieved the mastery standard by the end of the year. Residents scored highest in Leadership/Communication (2.67 out of 3). They scored lowest on Flow (2.41 out of 3). Ninety percent of residents felt that the experience prepared them for being an attending.

**Discussion:** A mastery learning-based supervising experience successfully allows residents to progressively acquire skills associated with running a busy ED. We plan to continue to evaluate the effectiveness of this intervention by examining the number of residents meeting the mastery standard as well as feedback from residents and faculty.

## Introduction

While the Accreditation Council for Graduate Medical Education (ACGME) mandates that training programs incorporate progressive and graduated responsibility for trainees ( *ACGME Common Program Requirements*), little guidance is provided about how to implement these responsibilities. To our knowledge, there are no disseminated resources for residencies to design and implement a clinical supervisory role for Emergency Medicine (EM) resident physicians.

An informal needs assessment at the University of Wisconsin EM residency program revealed that many successful residents had difficulty transitioning to a supervising role when they became a senior resident due to the sudden shift in responsibilities: residents were now responsible for attending-level tasks such as monitoring flow in the department, keeping junior residents on track, and managing an interdisciplinary team of physicians, nurses and techs. This new set of skills is very different than the skills that residents practice during their first two years of residency.

Based on the results of this needs assessment, a group of EM faculty involved with education leadership designed a new clinical supervisory experience to allow trainees to progressively master skills associated with running an ED. We felt that it was essential for learners to experience as many of the stresses that come with being an attending as possible to truly create the most valuable learning experience for them, from activating a stroke code, to reading EKGs, to answering questions from nursing, to taking EMS calls ( Friedman, Elinson and Arenovich, 2005). At the same time, it was important that an attending always be present and available to ensure safe patient care and allow timely performance feedback to take place.

We hypothesized that learners would benefit from deliberate practice through repetitive experience in this entirely new area of their clinical practice. Further, because of the difficult nature of these skills, we anticipated that residents would take varying amounts of time to acquire expertise in a supervising role. Therefore, we designed our experience in accordance with Bloom’s Theory of Learning for Mastery ( Bloom, 1968), which takes many of these ideas into account. Here, we present our framework for implementation of a graduated responsibility supervisory role using mastery learning principles.

This implementation framework is intended for residency program leadership who wish to institute a graduated, clinical supervisory role for resident physicians in the emergency department. While no specific knowledge base or skill set is required to implement this framework, potential adopters should be familiar with mastery learning principles. Additionally, while this experience could be adapted for a variety of clinical environments, close ties with a community site willing to accommodate senior residents is ideal.

## Methods

At the outset of the project, education leaders within the department defined a set of core principles and expectations for the supervising role that could be used to guide the creation of the experience as it evolved as well as provide consistent messaging to the residents ( Appendix A). Our overall guiding principle was that residents in this supervisory role should be independently performing as many of the tasks of an attending physician as feasible within our environment. We also jointly arrived at four domains of clinical practice that were felt to be most critical to success as a supervising physician - Leadership/Communication, Flow, Patient Care and Organization. A rubric was created with a 3-point Likert scale for each domain ( Appendix B) for a total of 12 total points available per shift, and the highest score in each category equal to an attending-level performance; this evaluation was to be completed by the attending and would be discussed with the resident at the end of every supervising shift for approximately 2-5 minutes. The mastery standard was defined via a consensus of education experts in our department as 23 of 24 possible points across any two shifts (1 shift with a perfect score across all domains, 1 shift with a perfect score in 3 out 4 domains).

As supervising in a busy academic center adds a large number of complicating factors which may distract from the task of learning the skill of running a department (e.g. multiple levels of learners, complex patients requiring multiple subspecialists, larger footprint, higher volume), we decided that the initial site for all of our learners to supervise would be one of our community sites, a 17,000 visit/year ED staffed by our faculty group, with only surgery and orthopedic specialty services along with hospitalists available on site. We felt that this lower volume site, staffed mainly with Advanced Practice Providers (APPs), would most closely mirror the environment that most residents would be practicing in as attendings in their first job after graduation. To accommodate new learners at the site in a supervisory role, we developed a standard set shift guidelines and objectives ( Appendix C) to guide expectations for faculty, residents, nurses and APPs, and distributed this document to each of these groups by email, at in-person meetings, and posted in the department. We dubbed this new role the PAT, for Pre-ATtending.

Our clinical schedule was able to accommodate each of our 12 PGY-3 residents completing 4 weeks of PAT shifts (16 shifts) over the course of the academic year (AY). Each resident was initially assigned two 1-week (four 10-hour shifts) immersive experiences with the idea that they would be a constant presence over the course of the week to allow them to develop relationships with staff, have consistent practice with supervisory skills, and the ability to incorporate feedback immediately. We were careful to select shift times that were both high-volume, but also balanced in terms of coverage with APPs and medical students so as to not stretch the resident to supervise too many providers at once. Halfway through the academic year, we looked at all resident evaluations to determine which residents met the mastery standard.

In the 2 ^nd^ half of the year (blocks 8-13), residents who successfully achieved the mastery standard were given the choice of completing their final two weeklong PAT experiences at the community hospital, the large academic, tertiary care hospital, or working as the physician in triage. The academic site and physician at triage roles allowed interested residents to practice supervising in a faster-paced, higher volume and higher acuity environments, while the community site allowed residents with an interest in community medicine the opportunity to continue to hone their supervisory skills in a learning environment matched to their future interests (See Appendix D and E for guidelines and objectives for these sites, also distributed to all staff at the respective sites). As supervising at an academic site includes the requirement of ensuring learners of multiple levels are being educated, a 5 ^th^ evaluation category, Teaching, was added to the end of shift evaluations for residents at the academic site only.

**Figure 1.**
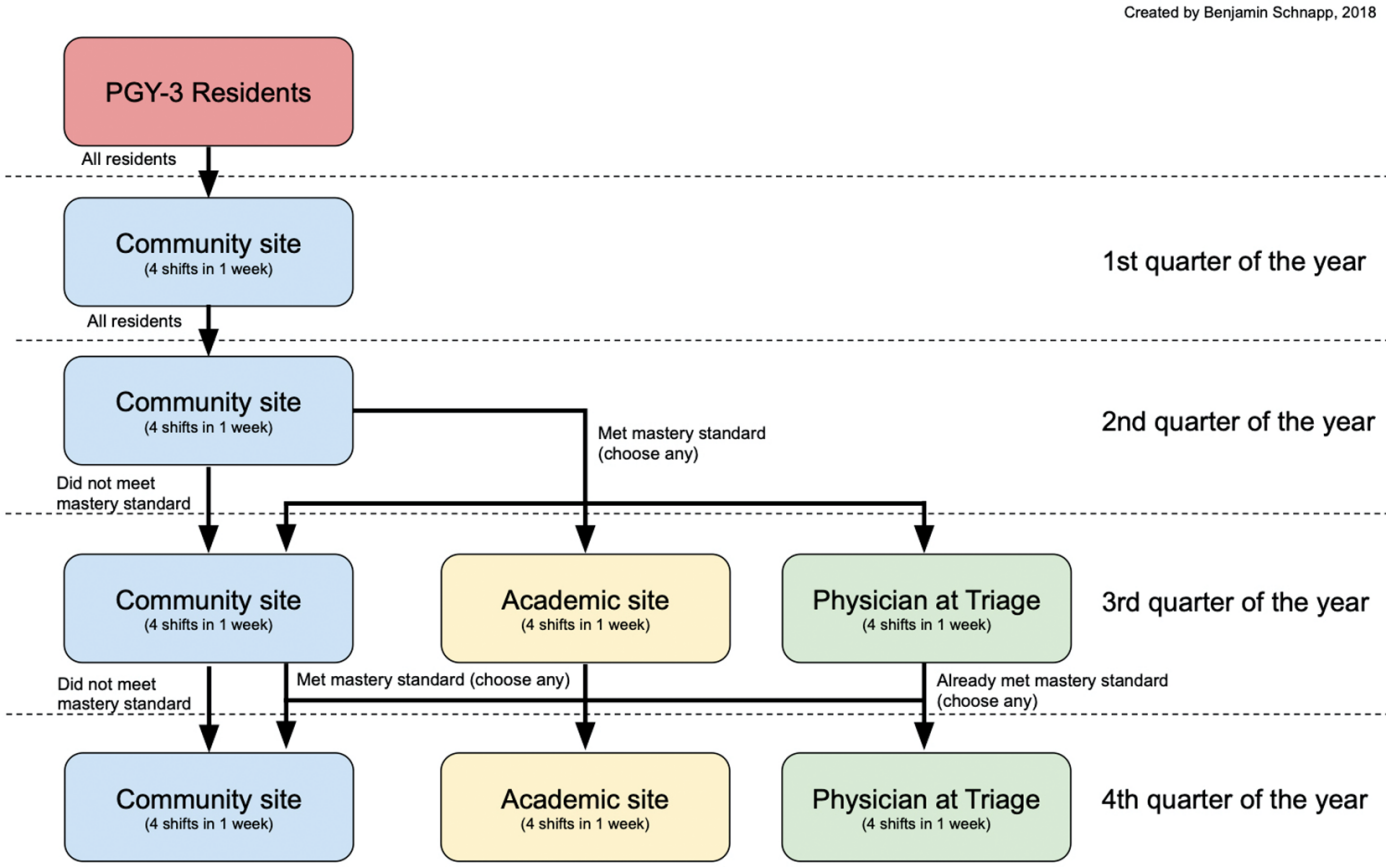
Clinical sites available for PAT supervision experience as residents progress through their PGY-3 year.

While residents were allowed to choose two different experiences for their final two weeks, they were required to participate at a selected site for a minimum of one week to allow them to iteratively improve their performance at the site. Residents who did not meet the mastery standard returned to the community site to continue to focus on refining their supervisory skills. After residents’ third week of supervising, we again looked at resident evaluations, and those who had newly met the mastery standard were allowed to choose any site for their final week. See Figure 1 for the options available to residents as they progressed through the year.

As we rolled out the new PAT role at each site, we wanted to ensure that all staff knew when a PAT resident was working, as well as what their responsibilities were, and the expectations they were expected to fulfill. In order make the PAT resident easily identifiable, we ordered each PGY-3 resident a set of red scrubs (not worn by any other staff in the department) and asked that they wear these only while working a PAT shift. When working, the PAT occupied the chair normally used by the attending to remind staff of their attending-like responsibilities. We also created a new provider column on our electronic medical record track board that clearly identified the patients that the PAT resident was taking care of. We held and attended numerous meetings, including nursing and tech staff meetings, faculty meetings, APP meetings, and ED leadership meetings. We also set aside time during weekly didactic conference to discuss the new role, answer questions and ensure psychological safety by emphasizing the challenging aspects of this new role and our faculty’s investment in improving residents’ practice through feedback. We posted advertisements ( Appendix F) in the ED, as well as break rooms and locker rooms to ensure all staff would be knowledgeable about the new role within the department. Time investment in the several months leading up to the start of the new supervising role averaged around 1 hour per week between attending various meetings, sending emails and posting advertisements, but was able to be split amongst multiple education faculty. We also performed brief, individual spot checks during the first few shifts of the PAT role at a new facility to rapidly identify and intervene on any problems that arose to prevent negative perceptions from taking hold.

While the above discusses the specifics of our implementation within our institution, we feel strongly that the success of the model is not dependent on these exact sites, and that the underlying principles would also work well in other clinical contexts. For example, introducing residents to supervising on lower volume overnight shifts could substitute for our community site, with residents who achieve mastery progressing to higher volume day shifts. Similarly, resident could start out supervising in a subset/pod of the department supervising interns only and progress to supervising a larger room with more levels of learners. Further, many of the other specific parameters of our intervention could also be easily adjusted to conform to the needs of the local institution. For example, an easily identifiable coat, stethoscope or badge backer could serve the same signifier role as the red scrubs served for us, residents could receive more or less supervising shifts as schedule demands allow, and the timing of the shifts could be adjusted to fit program needs (e.g. 2 consecutive weeks of supervising at a time instead of one).

To gauge the effectiveness of our intervention, we examined residents scores at all sites throughout the academic year 2017-2018, the number of residents who met the mastery standard, as well as the sites that residents who achieved mastery selected. We also sent a survey ( Appendix G) via electronic mail to all PGY-3 residents gauging their response to the new learning experience including how helpful they thought it was in preparing them for being an attending.

## Results

Overall, residents received the highest evaluations in the area of Leadership/Communication, averaging a 2.67 (out of a possible 3 points). Resident average scores were similar in the areas of Patient Care (2.54 out of 3) and Organization (2.52 out of 3). Average scores were lowest in the Flow category, 2.41 (out of 3).

Ten of 12 achieved mastery status at our community site and were allowed to choose their next supervising experience. Of these residents who met the mastery standard, 61% chose the academic site, 11% chose the Physician at Triage role, and 28% elected to remain at the community site.

Ten of the 12 (83%) PGY-3 residents responded to our survey. All respondents (10/10) Agreed or Strongly Agreed that the PAT experience was a good use of their time. Ninety percent of respondents (9/10) Agreed or Strongly Agreed that they felt more prepared to be an attending because of their PAT experience. Eighty percent (8/10) Agreed or Strongly Agreed that they learned things in the PAT role that they would not have otherwise. Seventy percent (7/10) Agreed or Strongly Agreed that feedback that they received in the PAT role helped them improve as a physician and 50% (5/10) Agreed or Strongly Agreed that the experienced helped them secure a job after graduation.

## Discussion

According to the ACGME, graded and progressive responsibility is “one of the core tenets of American graduate medical education” ( *ACGME Common Program Requirements*). It follows that residents in the final stages of training may carry a responsibility load approximating that of an attending physician. Our “pre-attending” or “PAT” role achieved its goal of allowing residents to develop supervisory skills by spending dedicated time on these skills and receiving targeted feedback in an environment that is conducive to pushing residents to the limits of their abilities, regardless of the pace at which they develop. Faculty agree that the level of independent practice demonstrated by PAT residents significantly exceeds that of PGY-3 residents who supervised in the department previously and survey results show that residents feel more prepared to an attending after graduation. Anecdotally, the shifts are also now advertised by residents as a significant strength of the program for preparing them for independent practice.

Prior to rollout of a mastery standard, we trialed a supervisory role at our academic site whereby every senior resident acted as a supervisor at our high volume/complexity site. During this trial, we found that faculty and residents tended to work as a pair, seeing patients, hearing presentations & engaging with consultants together. While efficient, enjoyable and somewhat educational, we found the PGY-3 resident to be over-supported in the role, which often amounted to mere shadowing of the attending. In order to allow the resident to develop independently and truly take on the larger load that progressive responsibility would dictate, we realized that the attending would need to function in the background. We sought feedback from faculty about why they preferred a side-by-side staffing model with residents, rather than allowing residents to work on their own. Significantly, we found faculty were not comfortable delegating a full attending load to every PGY-3 resident at our high volume/complexity site, as many residents struggled in this role. By moving to a mastery standard which identified residents who were ready to take on attending-level responsibility, we significantly increased faculty comfort and independence for PGY-3 supervising residents.

One factor that we felt was absolutely essential to the success of this program was clear expectations and frequent communication with all staff impacted by our change. The introduction of an entirely new role within a busy ED is extremely complex, especially one that had the potential to cause delays via the insertion of a new layer of bureaucracy (midlevel presents to PAT who then must present to attending). Messaging the role thoroughly to all stakeholders, with residency leadership attending various meetings to introduce the new workflows allowed us to directly answer questions that arose and avoided the spread of misinformation. We also supported this work by disseminating emails & posted flyers, as schedules of ED staff can be irregular and there was a need to ensure all staff were on board. Emphasizing the key educational benefits of increased supervising experience for our residents proved to be very effective at creating buy-in. We also felt that drafting, distributing and posting a practical reference guide for activities PATs can and cannot perform was critical for avoiding confusion and disagreements early on in the rollout and ensured smooth functioning when uncertainty arose with the new role.

An initial concern was that the mastery passing standard that had been set by consensus would not match faculty gestalt of supervisory performance, promoting residents too early or frustrating high-performing residents who were ready to take on a new challenge. On follow-up however, we found the discriminatory score for progression to the high volume/complexity site was acceptable to faculty and residents as it corresponded well to residents who were broadly felt to be performing well clinically and with supervising. For residents that did not meet the passing standard, we were able to use the evaluations from their PAT shifts to provide them with targeted improvement plans which residents found valid and actionable. We plan to continue to evaluate both the number of residents who meet the mastery standard as well as continuing to monitor the effectiveness of our passing standard moving forward to determine the need for a more formal standard setting exercise.

Our 2 clinical sites offer very different overall levels of volume and specialty care, thereby offering an intuitively appealing path for progressive responsibility. In many ways, however, some of the distinctions between our sites are arbitrary, as we find the lower volume/complexity site requires additional skills in managing surges of patients, as the department runs single physician coverage 24 hours a day, as well as interfacility level of care and transport considerations which are not present at the academic site. Despite this, these two sites appeared to work well for residents to progressively acquire supervision skills. There were initial concerns about whether EM residents would adversely affect efficiency by introducing a new layer of potential inefficiency at a site used to operating without residents ( Lammers *et al.*, 2003), however we have not observed a noticeable increase in ED length of stay. It remains to be determined however if our model is ideal for skills acquisition or if an alternative model (e.g. initially supervising a small pod of rooms at our academic site) would be superior.

## Limitations

Our data shows only the experience of one year’s worth of PGY-3 residents; it is possible that a different or larger group or residents would have a different experience with our intervention. It may also be subject to a novelty effect; resident enthusiasm may wane in subsequent years and logistical problems may develop without the benefit of the intensive oversight provided to a novel intervention. While we did not note significant effects on patient throughput, it is possible that an effect exists; this merits further study. We also did not collect extensive qualitative data for the purposes of our study; while the informal feedback we received on the PAT role was positive, a more extensive exploration of the strengths and weaknesses of the role with an unbiased interviewer remains an avenue for future work. Finally, this intervention was utilized only at one institution; while we hypothesize that the principles and procedures outlined here would work well in other, similar contexts, that has not been proven.

## Conclusions

As a competency-based education framework takes shape across the graduate medical education landscape, development of meaningful progression metrics for senior residents who are supervising will become increasingly important. While initiatives such as this one, as well as the McMaster Modular Assessment Program (McMAP) in Canada ( Chan and Sherbino, 2015) have made some progress towards this goal, more needs to be done to develop and validate metrics for supervising residents, with the eventual goal of creating a comprehensive and integrated framework of progression for all residents throughout their training program.

## Take Home Messages

• The ACGME mandates that physician training programs implement graduated responsibility.
• A supervising resident role can allow senior residents to safely perform many of the functions of an attending.
• A site with fewer complexities may allow residents new to supervising to progressively master new skills associated with the supervising role.
• The implementation of a new supervising resident role at a site that previously lacked trainees requires significant planning and coordination to make the transition as smooth as possible.
• Emergency medicine residents find an attending-level supervising experience valuable and educational.

## Notes On Contributors

**Benjamin Schnapp** is the Associate Program Director at the University of Wisconsin. He trained in Emergency Medicine at The Mount Sinai Hospital in New York City, where he completed a specialty track in medical education and served as chief resident. He completed his medical education fellowship at Northwestern University in Chicago and his Masters in Education with a focus on the Health Professions at the University of Cincinnati. ORCID ID: https://orcid.org/0000-0001-5031-8269

**Aaron Kraut** is the residency Program Director at the University of Wisconsin. He trained in emergency medicine at Northwestern University, serving as Chief Resident during his fourth year. He graduated from Middlebury College with a B.A. in Biology and completed medical school at the University of Vermont Larner School of Medicine.

**Ciara Barclay-Buchanan** is the Medical Director of the University of Wisconsin Emergency Department and Service Chief at the William S. Middleton VA Emergency Department. She completed her training in emergency medicine at Sinai-Grace Hospital in Detroit, Michigan where she served as a Chief Resident. She then served as the Associate Residency Director for the Emergency Medicine program at Sinai-Grace Hospital and at the University of Wisconsin before transitioning to an administrative role.

**Mary Westergaard** is the Vice Chair of Education for University of Wisconsin Emergency Medicine. After earning her medical degree from the Johns Hopkins University School of Medicine, Dr. Westergaard completed her emergency medicine residency training at Denver Health Medical Center, where she served as chief resident during her final year. Upon graduation, she moved back to Baltimore to join the faculty of the Johns Hopkins Emergency Medicine Residency Program where she cultivated an interest in resident and medical student education. Upon moving to the University of Wisconsin, she served as Assistant Director and then Director of Medical Student Education before serving as Residency Program Director from 2014-2019.

## Appendices

### Appendix A Overall Expectations and Principles

-We expect the achievement of extremely high standard (mastery) before progression to the next level of responsibility.

-No one is a master the first time they try something. Accordingly, no one should expect to achieve mastery level performance on your first PAT shift.

-Failure to achieve mastery standard is not a personal failing, but an opportunity for identifying areas for growth and improvement alongside your attending.

-Attendings are responsible for coaching residents on their identified deficiencies to give them the greatest opportunity to achieve mastery.

-8 shifts (2 blocks of 4 shifts) at the community site will be given to each resident as a starting point, but the pace to achieve mastery will be different for each resident. Some may achieve the standard after only a few shifts, others may take all year.

-There are no set quotas of residents with respect to the mastery standard. If all 12 residents meet the standard and progress, excellent; ideally all residents will be progressing towards mastery by the end of the year.

-Mastery will be judged to have been achieved when the PAT receives a combined score of at least 23 out of 24 possible points on twoindependent attending faculty evaluations for performance on two different shifts.

### Appendix B End of Shift Assessment Tool

Organization

1: Needs guidance on ordering of tasks.

Significant details may be lost.

Limited/poor utilization of staff.

2: Able to organize work adequately.

Details may be lost.

Occasional utilization of staff.

3: Tasks are ordered thoughtfully/optimally.

All details are accounted for.

Frequent utilization of staff.

Patient Care

1: One or more plans could result in a poor outcome.

Needs frequent reminders to reassess patients.

2: Plans adequate, minor adjustments may be needed.

May need occasional reminders to reassess patients.

3: Plans are excellent, need almost no adjustment.

Reassessments are completed independently.

Flow

1: Lags behind the flow of patients in the department.

Dispositions may be delayed.

Situational awareness needs improvement.

2: Able to keep up with average flow.

Could get overwhelmed with large volume of patients.

Mostly timely dispositions.

Situational awareness is moderate.

3: On top of all patients regardless of volume.

Consistent timely dispositions.

Situational awareness is excellent.

Leadership/Communication

1: Inadequate communication with team.

Patients often unclear on plan.

Looks to attending for team leadership.

2: Mostly good communication with team.

Patients sometimes aware of plans.

Usually serves as the team leader.

3: Always clear communication with team.

Patients always up to date on plan.

Always recognized as the team leader.

### Appendix C Community Site Workflow Guidelines

**Table:**

Responsibility	Role
Identification	PAT resident will be identifiable by red scrubs worn while on shift
Location	PAT resident will sit at the computer normally occupied by the attending Attending to use any other workstation during these shifts, per their preference
Patient introductions	PAT responsible for introducing/explaining team roles and terminology: -Attending physician -PAT Resident -APP -Medical student
Medical Students	Medical students to present to PAT on all patients PAT writes note on all student patients (may NOT copy student note) Attending may be present during student presentations to provide additional teaching
APPs	APPs to discuss their patients with PAT as they would with an attending Resident may NOT delegate patients as APP-only
Nurses	Nurses should communicate all care issues directly to the PAT (except in extenuating circumstances)
EKGs	PAT resident may be first reviewer, must immediately deliver to attending for review
Labs/Imaging	PAT resident should take all critical results from the lab or radiology
Culture Follow-Ups	PAT resident should field these inquiries and decide what course of action should be taken after discussion with the attending
Phone Calls	PAT resident will carry the primary attending phone and answer all phone calls, including from outside facilities. PAT resident must document all phone calls, and consult attending for any non-routine matters.
EMS Calls	PAT should listen to EMS calls when feasible and provide direction to prehospital providers.
Activations	PAT is responsible for all critical activations (STEMI, stroke, etc.) in consultation with the attending.
Floor Emergencies	PAT resident carries the code pager and responds to all pages.
Sign-out	PAT Resident initiates sign-out, ensures all parties are present PAT Resident signs out all patients to oncoming team.
Attending-only patients	Attending may pick up patients as needed per their assessment Attending does not present to PAT, but PAT should be aware of these patients’ one-liner
PAT Resident-Attending Communication	PAT resident will check in with attending at least 2-3 times per hour to run the list.
Attending-patient interaction	Attending MUST see every patient Timing: at attending convenience, ideally after PAT Resident has seen

### Appendix D Academic site workflow guidelines

**Table:**

Responsibility	Role
Identification	PAT resident will be identifiable by red scrubs worn while on shift
Timing	PAT residents shifts will occur from 7a-4p
Location	PAT resident will sit at the South side computer normally occupied by the attending Attending to use any other workstation during these shifts, per their preference
Patient introductions	PAT responsible for introducing/explaining team roles and terminology: -Attending physician -PAT Resident -APP/Junior resident/Student
Medical Students	Medical students to present to PAT on all patients PAT writes note on all student patients (may NOT copy student note)Attending may be present during student presentations to provide additional teaching
Advanced Practice Providers (APPs)	APPs to discuss their patients with PAT as they would with an attending
Nurses	Nurses should preferentially communicate *attending-level * care issues to the PAT
EKGs	PAT resident should be first reviewer, must immediately deliver to attending for review
Labs/Imaging	PAT resident should take critical results when primary provider is unavailable
Follow-Ups	PAT resident should field these decide with attending what course of action should be taken
Phone Calls	PAT resident does not take Access phone calls. All other phone calls can come to the PAT.
Medflight/EMS Calls	PAT should answer these if available, along with the attending.
Pediatrics/Traumas	PAT residents should see these simultaneously with attending to reduce communication issues
CDU	PAT residents should round in the CDU with the attending and assist in management as feasible
Activations	PAT is responsible for all critical activations (STEMI, stroke, etc.) in consultation with the attending.
Sign-out	PAT Resident initiates sign-out, ensures all parties are present, and signs out all patients
High volume workflow	Attending may pick up patients as needed per their assessment, ideally as delegated by PATAttending does not present to PAT, but PAT should be aware of these patients’ one-liner This should be viewed as an option of last resort
PAT Resident Communication	PAT resident will check in with attending and each team member 2-3 times per hour (new patient presentations and updates on old)
Attending-patient interaction	Attending *must* see every patient Timing: at attending convenience, ideally after PAT Resident has seen Attending should aim to allow PAT practice latitude when feasible
Attending-Junior Resident/Medical Student Interaction	Focused on teaching Should not ask Junior Resident to present 2 ^nd^ time.

### Appendix E Physician at Triage Workflow Guidelines

**Table:**

Responsibility	Role
Identification	PAT resident will be identifiable by red scrubs worn while on shift
Location	PAT resident will sit at the computer normally occupied by the attending Attending to use any other workstation during these shifts
Patient introductions	PAT responsible for introducing/explaining team roles and terminology: -Physician at Triage-APP-Team in the back
Triage	PAT will perform the triage function of the physician at triage, including:-Assigning patients to Main ED, Fast Care Area (FCA), MPP, or discharge from triage-Assigning Main ED patients a priority of high, medium or low.-Requesting immediate bed placement for critical patients from charge nurse
Orders	PAT resident responsible for placing appropriate orders on all patients triaged.
Flow	PAT resident responsible for FCA flow, including: -utilization of rooms, including MPP area -utilization of FCA/CDU APP, as appropriate
Start Up/Shut Down	Attending to be primarily responsible for navigating start up and shut down.
Advanced Practice Provider (APP)	FCA APP to discuss their patients with PAT as they would with an attending.
Nurses	Nurses should communicate all care issues directly to the PAT as feasible
EKGs	PAT resident may be first reviewer, must immediately deliver to attending for review
Labs/Imaging	PAT resident should take all critical results from the lab or radiology
Activations	PAT is responsible for all critical activations (STEMI, stroke, etc.) in consultation with the attending
Sign-out	PAT Resident signs out all remaining FCA patient to North attending at end of shift
Attending role	Attending may pick up patients as needed per their assessment Attendings may add or subtract orders placed by the PAT resident Attendings may override triage decisions made by the PAT resident
PAT Resident-Attending Communication	Attending will initially shadow the PAT resident If comfortable, attending can allow PAT resident to see patients independently Attending should aim to allow PAT practice latitude when feasible
Attending-patient interaction	Attending MUST see every patient assigned to FCA and discharged directly from triage Timing: at attending convenience

### Appendix G Supervising role survey

1. The PAT experience so far was a good use of my time.

Strongly Agree

Agree

Neutral

Disagree

Strongly Disagree

2. I have learned a lot in the PAT role that I would not have learned otherwise.

Strongly Agree

Agree

Neutral

Disagree

Strongly Disagree

3. I feel more prepared to be an attending next year because of the PAT role.

Strongly Agree

Agree

Neutral

Disagree

Strongly Disagree

4. The feedback I received in the PAT role helped me improve as a physician.

Strongly Agree

Agree

Neutral

Disagree

Strongly Disagree

5. My experience as a PAT helped me get a job for next year.

Strongly Agree

Agree

Neutral

Disagree

Strongly Disagree

6. Please provide any feedback for improvement of the PAT role going forward. (free response)
