# Supplementary material for: A Graduated Responsibility Supervising Resident Experience Using Mastery Learning Principles
Source: MedEdPublish (2016). 2020 Jun 30;8:203. Originally published 2019 Nov 14. [Version 2] doi: 10.15694/mep.2019.000203.2 (PMC10712466; doi:10.15694/mep.2019.000203.2)
Supplement: Peer review history

# Reviewer report: Reviewer response for version 2

**DOI:** 10.21956/mep.20254.r31425

**Published:** 2021-02-10

**Authors:** Richard Hays

**Affiliations:** 1 James Cook University

This review has been migrated. The reviewer awarded 4 stars out of 5 This paper reports an interesting innovation - a structured and supported transition program for senior residents who will soon be attending physicians. Transitions are often stressful, because responsibility can increase dramatically overnight. Here the emphasis is more on the leadership and supervision roles, while clinical competence is assumed at that level. Support is provided, thereby encouraging confidence-building in both residents and more senior staff. I agree that the authors have addressed the reviewers' comments on version 1.

Reviewer Expertise:

NA

No decision status is available

# Reviewer report: Reviewer response for version 2

**DOI:** 10.21956/mep.20254.r31424

**Published:** 2020-07-30

**Authors:** Ken Masters

**Affiliations:** 1 Sultan Qaboos University

This review has been migrated. The reviewer awarded 4 stars out of 5 The authors have met the main requirements stated in my review of their Version 1 of this paper, although I would like to have seen a split between the Agree and Strongly Agree because of the small sample. Nevertheless, a good tidying up of an already fine paper deserving a good read.

Reviewer Expertise:

NA

No decision status is available

# Reviewer report: Reviewer response for version 1

**DOI:** 10.21956/mep.19128.r27618

**Published:** 2020-02-08

**Authors:** Ken Masters

**Affiliations:** 1 Sultan Qaboos University

This review has been migrated. The reviewer awarded 4 stars out of 5 An interesting paper describing a graduated responsibility supervising resident experience, using mastery learning principles in an Emergency Department.The paper clearly outlines the central problems that residents are expected to transition to a supervisory role during their senior residency, but there is frequently little or no preparation for that phase, and so problems occur. A needs assessment then led to the development of the training programme. The implementation of the programme was well thought out, down to the smallest detail (e.g. red scrubs).Two areas of concern that I have:• The Results section is rather sparse – I would like to see raw figures as well as percentages. This is especially because, given N=12, it means that “Ninety percent” and “Eighty percent” etc. have been severely rounded off. Also, while combining “Agree”/”Strongly Agree” (and, similarly, “Disagree”/”Strongly Disagree”) is common in many studies, in a sample of this size, it could hide an important level of detail. So, perhaps a Table or two could be used.• The small sample size, although mentioned in the limitations, does impact on the ability to generalise. It would have been useful if the researchers had included qualitative questions, the answers to which would have assisted them and others in improvements. For future runs and evaluations, qualitative questions really should be included.Minor:• "APP" should be written out in full the first time it is used.Overall, the paper is well-structured and written, and the authors are to be commended for laying out the procedural details and guidelines in a manner that is easy to follow and assess.

Reviewer Expertise:

NA

No decision status is available

# Reviewer report: Reviewer response for version 1

**DOI:** 10.21956/mep.19128.r27619

**Published:** 2019-11-21

**Authors:** Felix Silwimba

**Affiliations:** 1 University of Lusaka

This review has been migrated. The reviewer awarded 5 stars out of 5 this study has addressed an important area in physician education. the competencies required for residents that they eventually build on as they grow in the practice of medicine. I recommend this article to be read by all medical educators including those in low-middle in come countries.

Reviewer Expertise:

NA

No decision status is available

# Reviewer report: Reviewer response for version 1

**DOI:** 10.21956/mep.19128.r27621

**Published:** 2019-11-18

**Authors:** Subha Ramani

**Affiliations:** 1 Harvard Medical School, Brigham and Women's Hospital

This review has been migrated. The reviewer awarded 5 stars out of 5 The authors have addressed an important area, i.e. professional transition from being a trainee to a position as a clinical supervisor. This is an innovative experience where supervision skills are learned towards the end of training rather than on the job. Transitions are anxious times and it is routinely expected that clinical professionals take on new roles without formal training. As the authors emphasize, the senior emergency dept resident is expected to supervise their junior peers, continue to develop their own knowledge and clinical skills, and manage an interprofessional team.They have adopted a mastery learning framework appropriately, stated clear study hypotheses, created a set of competencies that have developmental logic, observable and lead towards independent practice. Their methods are well described and appropriate to address their hypotheses. They have been creative in piloting the rotation at a community hospital, which may be less chaotic to study the impact of the program, yet ensured safety in patient care with attending presence. The authors have also communicated with all stakeholders (attending physician, interprofessional staff etc) about the objectives of this program, developed a robust formative assessment and created a remediation system within their program.I agree with their statement of limitations about implementation and evaluation of the program at different sites and further assessment of patient care quality. I would also recommend a qualitative arm to their study as this could reveal further benefits, challenges and opportunities that may not emerge from the survey alone. One last thing is to ensure psychological safe environment for all involved- the senior residents, junior residents, attending physicians and interprofessional staff.This paper will be very useful for all health professions educators, who might design a program dedicated towards essential skills that trainees need when they move into independent practice beyond clinical knowledge and skills.

Reviewer Expertise:

NA

No decision status is available

# Reviewer report: Reviewer response for version 1

**DOI:** 10.21956/mep.19128.r27620

**Published:** 2019-11-14

**Authors:** Carla Todaro

**Affiliations:** 1 Azienda Sanitaria Universitaria Integrata di Trieste

This review has been migrated. The reviewer awarded 5 stars out of 5 An excellent work on competency based education.This study has been designed with strong method giving attention to the different phases of the project development.The advantage for the trainees is to gain experience as "pre-attending" during residency and to become aware of the shift in responsibilities at attending level allowing them moreover to master learning outcomes and to increase their experience as professionals.The "end of shift assessment tool" proposed in this study, evaluating organization/leadership/flow/patient care is definitely a useful tool and a complete model, certainly it can be used in other programs for trainees assessment!

Reviewer Expertise:

NA

No decision status is available
